# Arginine methylation as a regulatory ratchet in cancer: From substrate selection to malignant‐state stabilization

**DOI:** 10.1002/1878-0261.70322

**Published:** 2026-08-25

**Authors:** So Hyun Kwon, Ji Min Lee

**Affiliations:** ^1^ Graduate School of Medical Science and Engineering Korea Advanced Institute of Science and Technology (KAIST) Daejeon Republic of Korea

**Keywords:** arginine methylation, cancer, post‐translational modification, protein arginine methyltransferase, regulatory ratchet

## Abstract

Arginine methylation, catalyzed by the protein arginine methyltransferase (PRMT) family, is a widespread post‐translational modification yet its regulatory logic in cancer remains incompletely framed. Unlike rapidly reversible modifications, arginine methylation operates through a persistence‐prone, forward‐biased logic shaped by the lack of a broadly acting demethylase system, multisite substrates, and ongoing methylation flux. Here, we propose a regulatory ratchet framework in which PRMT‐dependent methylation can reinforce cellular states through cumulative and network‐level effects rather than acting as a binary switch. We integrate PRMT subtype specificity and noncatalytic functions with a four‐layer model of substrate selection spanning sequence, structural, localization, and environmental context. Persistence may arise at both mark and flux levels, while scaffold‐like PRMT functions may sustain regulatory complexes independently of catalysis. In cancer, PRMT‐compatible substrates are distributed across RNA processing, chromatin and transcriptional regulation, signaling, and metabolism, with methylation output shaped by dynamic inter‐PRMT relationships. Together, these network‐level interactions may reinforce cancer cell states while creating therapeutic vulnerabilities within a constrained methylation system, shifting attention from individual PRMT abundance toward the network architecture that sustains malignant programs.

## Graphical abstract

Arginine methylation can be viewed as a persistence‐prone post‐translational modification regulated by a network of PRMTs. Competitive and compensatory interactions among PRMTs can redistribute methylation across substrate pools shaped by sequence, structural, spatial, and environmental layers, reinforcing RNA‐processing, chromatin, and signaling programs in cancer. This review explores how this regulatory ratchet may contribute to malignant‐state maintenance and therapeutic opportunities.

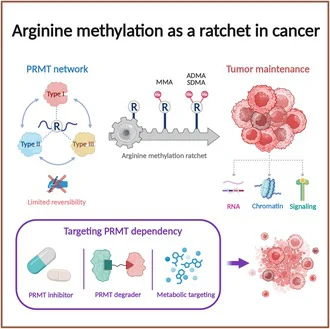

**Abbreviations**
ADMA: asymmetric dimethylarginine
AMPK: AMP‐activated protein kinase
CARM1: coactivator‐associated arginine methyltransferase 1
CDK1: cyclin‐dependent kinase 1
CRBN: cereblon
GAR: glycine‐ and arginine‐rich
hnRNP: heterogeneous nuclear ribonucleoprotein
IDR: intrinsically disordered region
MEP50: methylosome protein 50
MMA: monomethylarginine
MTA: methylthioadenosine
MTAP: methylthioadenosine phosphorylase
PRMT: protein arginine methyltransferase
PROTAC: proteolysis‐targeting chimera
PTM: post‐translational modification
RG/RGG: arginine‐glycine and arginine‐glycine–glycine
RNP: ribonucleoprotein
SAH: S‐adenosylhomocysteine
SAM: S‐adenosylmethionine
SDMA: symmetric dimethylarginine
SH3: Src homology 3
SMN: survival motor neuron
snRNP: small nuclear ribonucleoprotein
THW: threonine‐histidine‐tryptophan
VHL: von Hippel–Lindau

## 1 Introduction

Protein arginine methylation is an evolutionarily conserved post‐translational modification (PTM) in which methyl groups are added to arginine residues, thereby regulating the function of histone and nonhistone substrates. Since the identification and characterization of the protein arginine methyltransferase (PRMT) family, arginine methylation has been implicated in diverse biological processes, including transcriptional regulation, RNA metabolism, DNA damage responses, signal transduction, and metabolic control [1, 2, 3]. Early studies of histone arginine methylation, such as PRMT1‐mediated H4R3 methylation and coactivator‐associated arginine methyltransferase 1 (CARM1, also known as PRMT4)‐mediated H3 arginine methylation, indeed established an important basis for understanding PRMTs as chromatin‐associated regulators of gene expression [4, 5, 6]. Over the past two decades, advances in methylarginine proteomics and chemical enrichment strategies have substantially broadened this view. Large‐scale studies have shown that many arginine‐methylated proteins are nonhistone regulatory proteins, particularly RNA‐binding proteins, splicing factors, transcription‐associated factors, and proteins involved in dynamic protein–RNA or protein–protein interaction networks [7, 8, 9, 10]. These findings do not diminish the importance of histone arginine methylation, but rather indicate that PRMT biology extends beyond a predominantly chromatin‐centered framework. PRMTs are therefore increasingly viewed as regulators of molecular assemblies that coordinate RNA processing, chromatin organization, DNA repair, signaling, and metabolic adaptation [2, 3, 11, 12, 13].

This expanded substrate landscape raises an important question for cancer biology: How do PRMTs generate context‐specific effects across such diverse regulatory systems? Recent studies suggest that the methylation output of PRMTs is shaped not only by enzyme subtype or catalytic activity but also by substrate architecture, local concentration, cellular compartmentalization, metabolic state, and disease‐specific context [8, 14, 15, 16, 17]. This perspective is particularly relevant in malignant cells, which rely on plastic and stress‐responsive programs coordinated by RNA‐processing factors, chromatin regulators, transcriptional cofactors, and signaling and metabolic pathways [18, 19, 20, 21]. Many of these proteins are established PRMT substrates or belong to substrate classes that are structurally or contextually compatible with PRMT engagement [2, 8, 11, 22]. Accordingly, PRMT dependency in cancer may reflect more than enzyme overexpression or the activity of individual oncogenic substrates. Rather, it may arise from how arginine methylation is allocated across substrate pools, how PRMT pathways compete, compensate, and cooperate with one another, and how methylation‐supported networks contribute to the maintenance of malignant cell states under proliferative, metabolic, or therapeutic stress [16, 23, 24].

In this review, we synthesize recent advances in PRMT biology to develop a state‐oriented framework for understanding arginine methylation. We first summarize the biochemical classification and structural basis of PRMT specificity. We then discuss arginine methylation as a forward‐biased regulatory system and propose a multilayered model that incorporates sequence‐level, structural, spatial, and environmental determinants of PRMT substrate selection. Moreover, we explore noncatalytic PRMT functions and their implications for therapeutic targeting, with particular attention to targeted protein degradation. Finally, we discuss cancer‐specific patterns of substrate allocation and inter‐PRMT relationships, and consider how these features may give rise to therapeutic vulnerabilities.

## 2 Biochemical basis and classification of arginine methylation

Arginine methylation is one type of PTM in which one or two methyl groups are added to the terminal (ω) nitrogen atoms of the guanidinium group of arginine [2]. Subsequent enzymological and molecular studies demonstrated that such methylation states are produced by a family of related but distinct enzymes rather than a single methyltransferase, leading to the definition of the PRMT family [25, 26]. Like other forms of protein methylation (e.g., lysine, histidine, glutamate, and aspartate), this process relies on S‐adenosylmethionine (SAM) as the primary methyl donor, which is synthesized from methionine and adenosine triphosphate by methionine adenosyltransferase [1, 27, 28]. Based on the number and distribution of methyl groups, three chemically distinct forms of methylarginine are recognized in mammalian cells: monomethylarginine (MMA), asymmetric dimethylarginine (ADMA), and symmetric dimethylarginine (SDMA) [29, 30].

At the reaction level, arginine methylation can proceed in a stepwise manner: MMA is formed first, and this intermediate can subsequently undergo a second methylation to generate a dimethylated derivative [31, 32]. This two‐step process is generally distributive rather than processive. Kinetic studies have shown that the monomethylated intermediate is released from the enzyme and rebinds before the second methyl transfer occurs [33, 34]. Such a release‐and‐rebinding step creates a kinetically discrete point where a monomethylated substrate may become accessible to alternative PRMTs, providing a potential mechanistic basis for inter‐PRMT substrate competition.

A key organizing principle of the PRMT family is that its members are classified according to the methylarginine products they generate through this reaction sequence [1]. Although all PRMTs can catalyze the initial monomethylation step, PRMT7 generates MMA as its terminal product and is classified as a Type III PRMT, demonstrating that this modification can serve as an endpoint rather than a mere intermediate [32, 35]. Meanwhile, the second methylation step introduces a major divergence. ADMA is formed by repeated methylation of the same terminal nitrogen atom, and that is catalyzed by Type I PRMTs consisting of PRMT1, 2, 3, 4, 6, and 8. By contrast, SDMA is generated by methylation of the other terminal nitrogen, which is carried by Type II PRMTs composed of PRMT5 and 9 [32, 35]. Nine canonical mammalian PRMTs have been characterized, each displaying distinct substrate preferences, subcellular localization, and regulatory properties [1, 25, 36].

Together, these findings establish that the PRMT family generates distinct methylarginine products through a shared catalytic framework, while differences in methylation outcome contribute to the functional diversity of PRMT‐dependent regulation.

## 3 Structural diversity and substrate specificity in the PRMT family

In PRMTs, conserved catalytic features and subtype‐specific structural variations together shape methylation outcome and substrate specificity.

### 3.1 Catalytic domain of the PRMT family

The PRMT family shares a highly conserved catalytic architecture built upon an N‐terminal Rossmann fold and a C‐terminal β‐barrel domain. These two structural modules together form the catalytic cleft at their interface, where the SAM and the substrate arginine are positioned in close proximity for methyl transfer [14, 37, 38].

Within the Rossmann fold, Motif I and the adjacent Post‐I motif form the binding pocket for SAM, stabilizing and orienting the methyl donor for catalysis. Meanwhile, Motifs II and III form conserved β‐sheet interactions that contribute to the organization and structural integrity of the catalytic fold. Elements within the catalytic cleft, including the double glutamate (E) motif and the threonine–histidine–tryptophan (THW) loop, contribute to functional specificity. The double E motif, composed of conserved acidic residues, orients the guanidinium group of the substrate through electrostatic interactions, whereas the THW loop contributes to substrate recognition and organization of the active site. As a whole, these six motifs define the catalytic mechanism of PRMTs by coordinating cofactor binding, substrate positioning, and SAM‐dependent methyl transfer [14, 38].

Despite this conserved catalytic framework, PRMT subtypes exhibit critical structural divergence within the catalytic cleft, particularly in the geometry of the substrate‐binding pocket. Here, divergence has been attributed to variations in key active‐site elements, including the double E motif and THW loop, as well as surrounding residues that collectively shape substrate positioning and catalytic geometry [2, 39, 40, 41]. These structural features, in turn, determine how the target arginine is oriented relative to the SAM cofactor, thereby controlling which terminal nitrogen is accessible for methyl transfer. Importantly, these conformational differences underlie the distinct catalytic behaviors and methylation outcomes of Type I and Type II PRMTs. In Type I enzymes, steric restriction on the one side of the arginine favors repeated methylation of the same terminal nitrogen, resulting in ADMA formation. Conversely, in Type II enzymes, an alternative pattern of spatial restriction favors access to the other terminal nitrogen during the second methyl transfer, enabling SDMA formation. Unlike other subtypes, PRMT7 presents more extensive structural constraints within the active site, which appear to prevent productive accommodation of monomethylated substrate for the second methylation event, thereby limiting the reaction to MMA formation. Consistent with this, comparative structural analyses across PRMT family members indicate that even minor alterations in these regions can shift product specificity, supporting the view that methylation outcome is encoded by specific features of active‐site architecture [42].

Taken together, these findings suggest that PRMT product specificity arises from a conserved catalytic framework combined with subtype‐specific structural adaptations, with the geometry of the catalytic cleft playing a central role in determining methylation outcome.

### 3.2 Noncatalytic domain of PRMT family

**Fig. 1:**
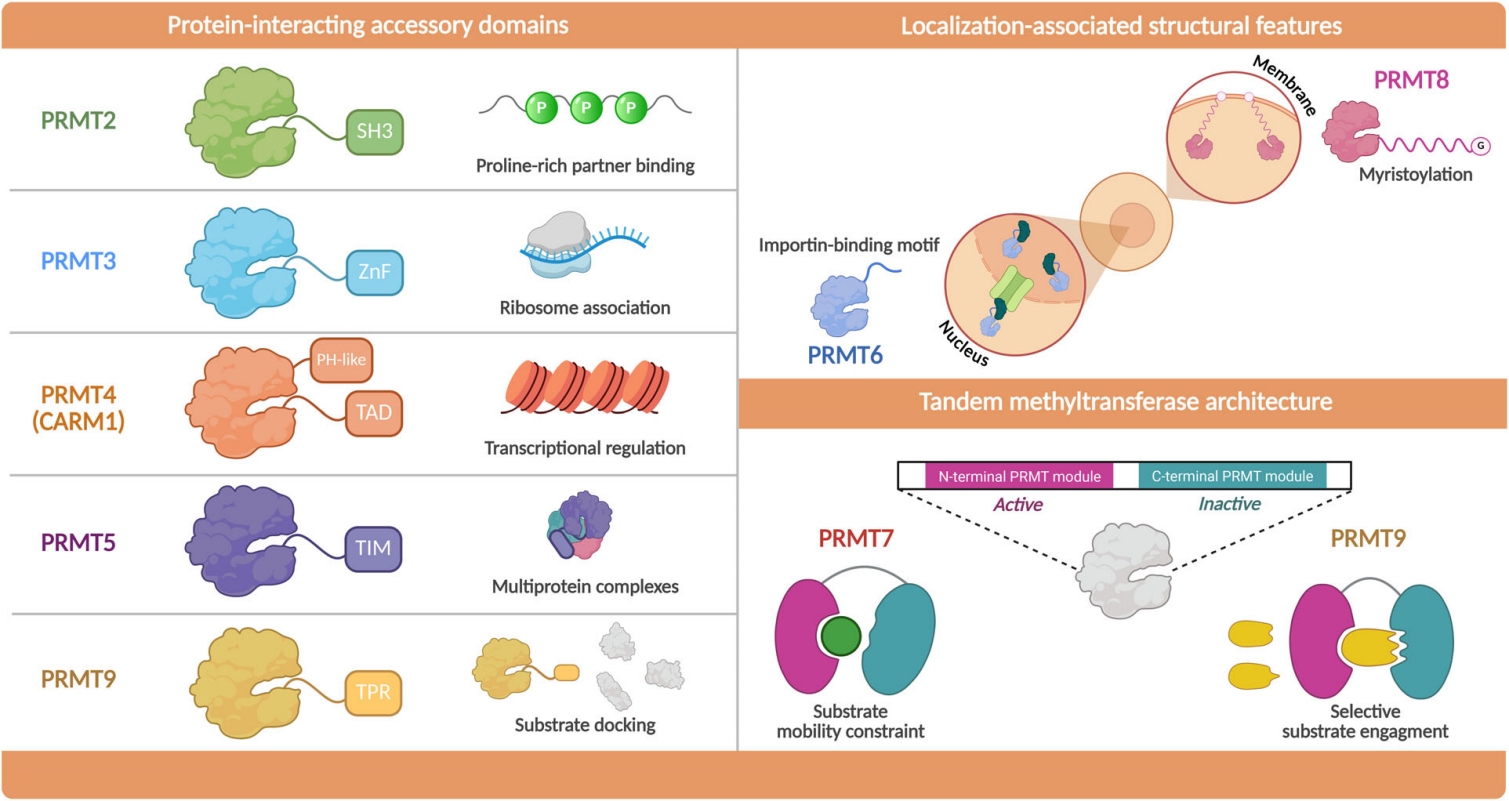
Subtype‐specific structural features diversify PRMT family function. The schematic summarizes representative structural features that contribute to functional specialization across PRMT family members. Accessory domains support protein interactions, complex assembly, and substrate engagement, whereas localization‐associated features direct PRMTs to distinct cellular compartments. Tandem architectures in PRMT7 and PRMT9 further shape substrate recognition, particularly by constraining substrate accommodation or supporting substrate engagement. CARM1, coactivator‐associated arginine methyltransferase 1; G, glycine; P, proline; PH, pleckstrin homology; PRMT, protein arginine methyltransferase; SH3, Src homology 3; TAD, transcriptional activation domain; TIM, triosephosphate isomerase; TPR, tetratricopeptide repeat; ZnF, zinc finger. Created with BioRender.com. https://biorender.com/6qvn9t1.

Despite sharing a conserved catalytic core, PRMTs exhibit considerable structural diversity outside this region. PRMT1 represents a minimal architectural framework, being largely confined to the conserved catalytic core [39]. In contrast, other PRMT family members have acquired additional structural elements that expand their functional repertoire, enabling diversification in interaction networks, subcellular localization, and substrate specificity (Fig. 1).

Multiple PRMTs harbor accessory domains at either N‐ or C terminus, which function as structural platforms for protein–protein interactions. PRMT2 is distinguished by an N‐terminal Src homology 3 domain formed by five antiparallel β‐strands, which creates a compact interaction surface for proline‐rich ligands and therefore broadens the protein–protein interaction repertoire of the enzyme [43, 44, 45, 46]. Similarly, PRMT3 harbors a Cys2‐His2‐type zinc finger domain that mediates interactions with ribosomal protein S2, directing the enzyme to ribosome‐associated substrates [47, 48]. Unlike other subtypes that possess auxiliary modules at the N terminus, CARM1 is distinguished by a C‐terminal transcriptional activation domain, which facilitates interactions with transcriptional coactivators and nuclear receptor complexes, thereby coupling arginine methylation to transcriptional regulation [49, 50]. Meanwhile, CARM1 also contains an N‐terminal pleckstrin homology‐like domain, providing an accessory structural module that affects protein–protein interactions and coactivator‐associated functions [38, 51]. PRMT5 contains a triosephosphate isomerase barrel—composed of eight alternating α‐helices and β‐strands—that supports homodimer formation and mediates interaction with methylosome protein 50 (MEP50), ultimately assembling a catalytically active hetero‐octameric complex with greater enzymatic activity than PRMT5 alone [40, 52, 53]. An extended helical scaffold in PRMT9 is known as tetratricopeptide repeat, whose characteristic interaction surface is thought to contribute to selective recognition and engagement of substrate or partner proteins [54, 55].

Beyond the incorporation of accessory domains that mediate molecular interactions, additional layers of structural variation among PRMTs contribute to differences in subcellular localization and catalytic organization. Similar to PRMT1, PRMT6 exhibits a compact structural framework and harbors a putative importin‐binding motif near the N terminus, which may contribute to its predominant nuclear localization [56, 57, 58]. PRMT8 contains an N‐terminal myristoylation site that has been reported to promote its association with the plasma membrane, providing a potential mechanism for spatial regulation of the enzyme [59]. PRMT7 and 9 are structurally distinct in that they contain two tandem methyltransferase domains within a single polypeptide, yet these duplicated modules contribute differently to enzyme function. In PRMT7, only the first methyltransferase domain is catalytically active, whereas the second domain is devoid of cofactor‐binding capacity and is therefore incapable of methyl transfer [32, 46, 60]. Instead, this inactive C‐terminal module contributes to a structurally constrained catalytic architecture at the N‐terminal active site, which may limit productive accommodation of monomethylated substrates for a second methyl transfer and therefore favor MMA formation. Meanwhile, the C‐terminal methyltransferase module of PRMT9, although catalytically inactive, is thought to fold together with the catalytically competent N‐terminal module to support productive substrate engagement [54, 61].

Collectively, these observations indicate that noncatalytic domains and higher order assemblies are not merely structural appendages but serve as critical determinants of substrate specificity. In this context, PRMT‐mediated methylation is shaped by an integrated mechanism in which the catalytic pocket defines the chemical reaction, whereas interaction modules and cellular context govern substrate selection.

## 4 Arginine methylation as a forward‐biased molecular ratchet

**Fig. 2:**
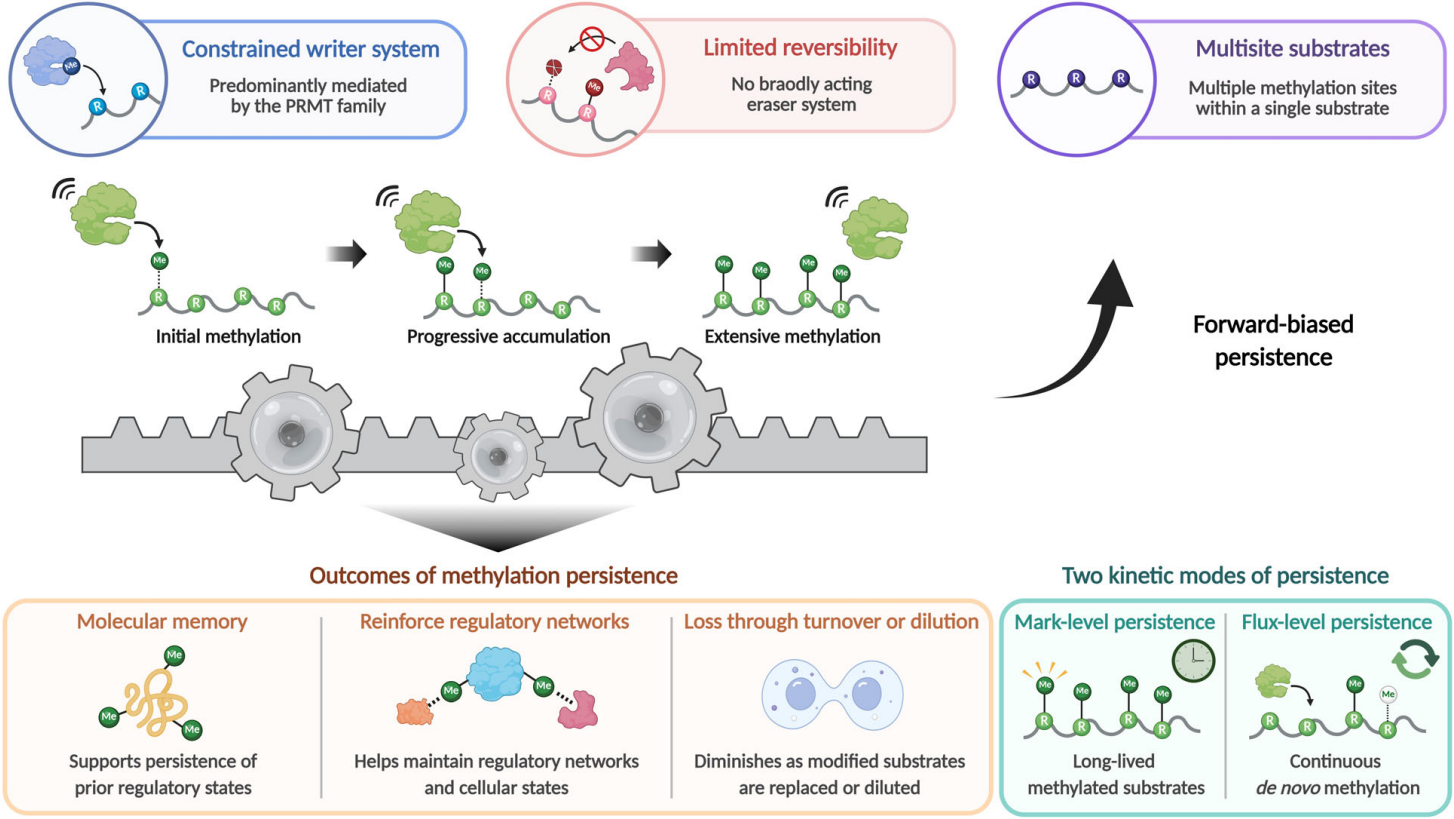
Mechanistic and kinetic basis of the forward‐biased arginine methylation ratchet. The schematic illustrates how a constrained PRMT writer system, limited reversibility, and multisite substrate architecture may favor progressive arginine methylation and persistence. Persistent methylation may contribute to the maintenance of regulatory states, while methylarginine signals may decline as proteins turn over or are diluted during cell division. Such methyl mark maintenance may arise at the mark level through long‐lived substrates or at the flux level through continuous *de novo* methylation. Me, methyl group; PRMT, protein arginine methyltransferase; R, arginine residue. Created with BioRender.com. https://biorender.com/3v1dox5.

Many regulatory PTMs are conceptualized as dynamic regulatory systems that enable rapid and reversible modulation of protein function, often through coordinated writer‐eraser enzyme pairs for oscillatory switching between functional states [62, 63]. Within this framework, phosphorylation and acetylation exemplify reversible signaling modalities that allow proteins to rapidly respond to fluctuating cellular inputs [64, 65]. By comparison, arginine methylation operates under more asymmetric regulatory constraints, potentially biasing the system toward persistence rather than rapid enzymatic reversal (Fig. 2).

### 4.1 A constrained enzymatic writer architecture

This distinction emerges from both the enzymatic architecture and the limited reversibility of the system. In mammalian cells, most characterized protein arginine methylation is mediated by the PRMT family, a structurally conserved but functionally diversified group of SAM‐dependent enzymes [3]. Canonical protein arginine methylation is concentrated within a relatively small PRMT family whose members display distinct substrate preferences and catalytic outputs [1]. This relatively constrained writer repertoire may limit the routes through which arginine methylation patterns are established and redistributed.

### 4.2 The absence of a generalizable arginine demethylase system

Equally important is the absence of a robust and generalizable ‘eraser’ system analogous to those governing lysine methylation, phosphorylation, or acetylation [64, 65, 66]. JMJD6 was first reported nearly two decades ago to function as a histone H3R2/H4R3 arginine demethylase, with supporting evidence from both purified enzyme preparations and cellular experiments [67]. However, this activity could not be reproduced in independent biochemical studies, which instead reassigned its principal catalytic function to lysyl 5‐hydroxylation of splicing regulatory proteins for U2AF65 [68]. Meanwhile, reports of JMJD6‐dependent loss of methylarginine on specific substrates have nonetheless continued to emerge, including on the estrogen receptor ERα in breast cancer cells and the stress‐granule protein G3BP1 [69, 70]. Yet, it remains unclear whether such substrate‐specific loss of methylarginine reflects direct demethylase activity or instead arises through indirect changes in methylation status.

Beyond JMJD6, several Jumonji C domain enzymes—whose canonical role is to remove methyl marks from lysine—have been implicated in arginine demethylation on selected substrates. While early evidence for arginine demethylase activity in this family was confined to *in vitro* assays using isolated peptides, recent findings highlight genuine cellular arginine demethylation events with defined physiological consequences [71]. JMJD1B has been reported to act on a histone methylarginine mark relevant to hematopoietic stem/progenitor cell development, whereas KDM5C demethylates the autophagy regulator ULK1 [72, 73]. These cases illustrate relatively substrate‐restricted arginine demethylation within defined biological pathways. KDM4A may represent a broader example, acting on multiple methylarginine substrates during mitotic progression—including histone mark H3R17me2a and the nonhistone PI3KC2α—as well as the metabolic enzyme IDH2 in mitochondrial metabolism together with KDM3A [74, 75, 76]. A further example is Mina53, whose demethylase activity has been linked to defined substrates and biological outcomes in more than one context. Mina53‐mediated demethylation of a histone methylarginine mark contributes to neural stem/progenitor cell regulation, whereas demethylation of p53 suppresses its signaling and promotes tumor growth [77, 78].

Two further mechanisms can reduce methylarginine signals without true erasure. Peptidylarginine deiminase 4 has been reported to convert mono‐ but not di‐methylarginine to citrulline, through a process called demethylimination [79, 80]. Later kinetic studies, however, showed that this reaction is far slower than citrullination of unmodified arginine and questioned whether it occurs efficiently *in vivo* [81, 82]. Even when it occurs, demethylimination does not regenerate unmodified arginine; it produces citrulline, a chemically distinct terminal residue that cannot serve as a reusable PRMT substrate. Separately, JMJD5 and JMJD7 remove methylarginine signals through proteolytic processing rather than demethylation. These enzymes cleave histone tails bearing methylated arginine and promote degradation of the released fragment, thereby eliminating the modified region of the substrate itself [83]. This represents specialized substrate turnover rather than enzymatic erasure.

Taken together, this expanding but still individually narrow set of candidate mechanisms has not converged on a broadly acting, substrate‐general reversal system comparable to canonical lysine demethylation. Methylarginine marks may persist until the modified protein is removed through protein turnover or dilution during cell division, unless acted upon by one of these context‐specific removal mechanisms. This view frames arginine methylation as persistence‐prone and forward‐biased, rather than absolutely irreversible, reflecting the current state of the field.

### 4.3 Multisite substrate architecture and state persistence

The potential for sustained methylation may be further influenced by the multisite nature of PRMT substrates. Arginine methylation is highly enriched within arginine‐glycine and arginine‐glycine–glycine (RG/RGG) motifs, which are frequently repeated within intrinsically disordered regions (IDRs) of RNA‐binding proteins [84, 85]. This sequence organization enables multisite methylation, allowing individual PRMTs to deposit modifications across multiple arginine residues within a single polypeptide. Proteomic analyses further reveal that arginine methylation is widespread and often occurs in a distributed manner across protein domains, rather than being confined to a single regulatory site [12]. As a result, methylation can accumulate progressively, generating a graded rather than binary modification landscape. Notably, recent work has shown that PRMT1 can form higher‐order oligomers that influence catalytic efficiency and substrate specificity, suggesting that oligomeric organization may provide an additional layer of regulation for multisite substrates [86].

Integratively, these features may support a graded methylation landscape that deviates from the classical paradigm of reversible PTMs and allow arginine methylation to encode a form of molecular memory. Within this framework, successive methylation events may progressively reinforce selected protein‐interaction networks and cellular states while reducing the capacity for rapid resetting [2, 3].

### 4.4 Mark‐level versus flux‐level persistence in the ratchet

Although the absence of a broadly acting eraser makes methylarginine comparatively persistence‐prone, the functional consequence of this persistence depends on the kinetic level at which the ratchet operates.

First, methylation can directly alter substrate turnover by increasing or decreasing the stability of the modified protein. A representative example is KLF4, whose PRMT5‐mediated methylation antagonizes von Hippel–Lindau (VHL)‐dependent ubiquitylation and extends KLF4 half‐life [87]. Conversely, disruption of this modification reduces KLF4 accumulation and compromises genome stability and cell‐cycle checkpoint responses [87]. A similar stabilizing relationship is observed for EZH2, in which a non‐methylatable mutant is degraded substantially faster than wild‐type EZH2, as PRMT1‐mediated methylation at R342 limits cyclin‐dependent kinase 1 (CDK1)‐dependent phosphorylation and subsequent TRAF6‐mediated ubiquitylation [88]. A follow‐up study further showed that the same methylation event blocks AMP‐activated protein kinase (AMPK)‐mediated phosphorylation, facilitates EZH2‐SUZ12/PRC2 assembly, and enhances breast cancer cell growth and tumor formation *in vivo*. Accordingly, xenograft tumors expressing the nonmethylatable EZH2 mutant grow substantially more slowly than those expressing wild‐type EZH2 [15]. Taken together, these findings link the same EZH2 methylation event to both protein stabilization and tumorigenic output, illustrating how persistence of a substrate‐level mark may reinforce a malignant regulatory state in a ratchet‐like manner. This stability logic is not invariably positive, however. In the case of PRMT5‐mediated methylation of ALKBH5, methylation instead promotes TRIM28‐dependent ALKBH5 degradation and ultimately increases CD276‐mediated immune evasion in colorectal cancer [89]. Thus, arginine methylation can act as a bidirectional regulator of substrate stability, with the direction of the effect determined by the specific PRMT‐substrate pair.

Second, persistence of the methylated landscape may also reflect continuous substrate synthesis coupled to efficient *de novo* methylation. PRMT5‐dependent methylation of Sm proteins provides an example of this flux‐dependent regulation. Rather than stabilizing individual Sm proteins, PRMT5‐dependent methylation strengthens SMN binding and thereby promotes Sm‐core assembly during snRNP biogenesis [90, 91]. Since newly synthesized Sm proteins continuously enter this pathway, ongoing methylation can maintain an assembly‐competent pool without requiring reversal of marks on pre‐existing substrates [92]. Similar logic may apply to other PRMT‐regulated processes that depend on repeated recognition of newly deposited methylation events. In this regime, persistence depends less on the lifetime of individual marks than on continuous methylation flux, allowing catalytic inhibition to reduce the methylated pool as substrate turnover proceeds even in the absence of a dedicated demethylase. Consistent with this, NMR‐based tracing of methylarginine turnover indicates that global arginine methylation and demethylation are slow processes in cells, particularly compared with phosphorylation [93]. Taken together, the ratchet may arise either from long‐lived methylated substrates or from continuous maintenance of short‐lived methylated pools.

This distinction also helps reconcile the ratchet model with the timing of phenotypic responses observed after pharmacological PRMT inhibition. Where such responses emerge before global methylarginine signals are maximally depleted, they need not imply complete reversal of a pre‐existing methylation landscape. PRMT inhibition may instead interrupt ongoing methylation‐dependent flux or push cells across threshold‐dependent fate boundaries. In this view, differentiation or apoptosis represents a transition into a new cellular state rather than simple restoration of the pre‐inhibited one. Conversely, when depletion of the pre‐existing methylated pool proceeds slowly, downstream responses may unfold on a similarly extended timescale. For instance, in MTAP‐deleted xenografts, MRTX1719 produced a progressive reduction in global PRMT5‐dependent SDMA, with partial suppression after 3 days and maximal inhibition after 14 to 21 days. Cell‐cycle‐associated changes emerged across this interval, whereas apoptotic markers became prominent at later time points coinciding with maximal pathway inhibition [94]. Taken together, the relative timing of methylarginine depletion and phenotypic transition is not fixed, but may depend on the kinetic regime governing a given PRMT‐regulated system.

## 5 A four‐layer model of PRMT substrate selection in the catalytic context

**Fig. 3:**
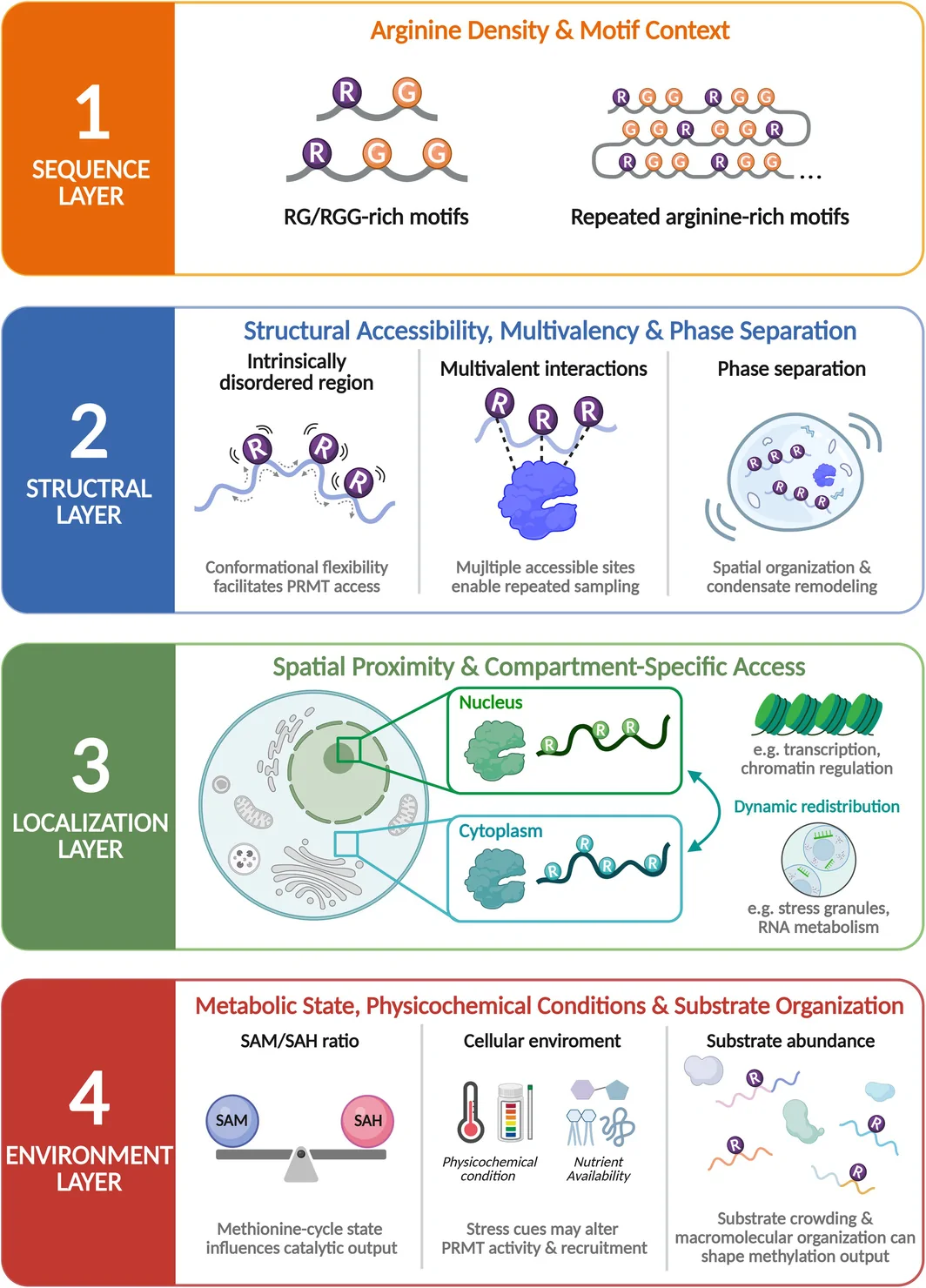
Multilayered determinants of PRMT substrate selection. Schematic model of four interconnected layers that shape PRMT substrate selection. Sequence context, structural accessibility and multivalency, subcellular localization, and environmental conditions collectively influence PRMT‐substrate engagement and methylation output. Together, these layers provide a framework for understanding context‐dependent substrate selection by PRMTs. G, glycine residue; PRMT, protein arginine methyltransferase; R, arginine residue; RG/RGG, arginine‐glycine and arginine‐glycine–glycine; SAH, S‐adenosylhomocysteine; SAM, S‐adenosylmethionine. Created with BioRender.com. https://biorender.com/eb0njrr.

To provide a conceptual framework for PRMT substrate selection, we organize the factors discussed below into four interconnected layers: sequence features, structural accessibility, subcellular localization, and environmental context (Fig. 3). Together, these layers may help explain how potential PRMT substrates are differentially engaged across cellular contexts.

### 5.1 Sequence layer—Arginine density and motif encoding

Arginine methylation is first shaped by primary sequence, where residue composition and motif organization define the landscape of potential modification. An early biochemical study suggested that glycine‐ and arginine‐rich (GAR) sequences, which are common in RNA‐binding proteins, can serve as efficient substrates for arginine methyltransferases [84]. A subsequent motif‐centered analysis further established RG/RGG motifs as recurrent sequence features of many arginine‐methylated proteins [85]. In this context, glycine residues adjacent to the target arginine are thought to increase local conformational flexibility, thereby facilitating access of the arginine side chain to the PRMT active site [66].

Nevertheless, these motifs should not be interpreted as strict consensus codes but instead individual PRMTs exhibit distinct but permissive sequence preference. Although PRMT1 frequently methylates GAR/RGG‐rich substrates, substrate profiling has revealed that glycine residues adjacent to the target arginine are not obligatory, supporting a broader model of PRMT1 substrate recognition beyond the classical RGG paradigm [95]. Similarly, PRMT6 displays broad specificity, preferring either an RG motif or basic and bulky residues around the target arginine [96]. In contrast, CARM1 shows a more distinct preference for proline‐containing motifs, as indicated by global substrate mapping and peptide‐array analyses [97, 98]. PRMT7 represents a more restricted case, preferentially targeting RxR motifs within lysine‐ and arginine‐rich regions [99].

Collectively, these examples indicate that PRMT substrate recognition is shaped by enzyme‐specific sequence biases rather than dictated by a universal consensus motif. Regardless, GAR contexts remain a recurrent feature across many PRMT substrates, indicating that these motifs provide a permissive but not absolute sequence environment for methylation. Thus, the sequence layer defines where arginine methylation is likely to occur, but it does not fully determine whether a given arginine residue will be modified.

### 5.2 Structural layer—IDRs, Multivalency, and phase separation

Whereas the sequence layer defines where methylatable arginine residues are encoded, the structural layer determines whether these residues are physically accessible and dynamically presented for PRMT engagement. Indeed, the presence of an arginine‐containing motif alone does not ensure methylation, because target residues must remain solvent‐exposed, conformationally available, and compatible with productive entry into the catalytic pocket.

From a structural perspective, intrinsic disorder provides a permissive context for arginine methylation by keeping target arginine residues conformationally flexible and dynamically accessible to PRMTs. This is particularly relevant for RNA‐binding proteins and chromatin‐associated regulators, many of which contain RG/RGG‐rich low‐complexity regions that are frequently targeted by arginine methyltransferases [2, 84, 85]. In such flexible regions, target arginine residues may more readily sample conformations that allow productive positioning of the guanidino group within the PRMT catalytic pocket. This interpretation is consistent with structural and mechanistic studies showing that methyl transfer requires precise alignment of the substrate arginine through conserved active‐site elements, including acidic residues and the THW loop [14, 38]. Importantly, IDRs are not only permissive before methylation but also responsive after it. Specifically, modification of RG/RGG arginines with methyl marks can weaken cation‐π and other multivalent interactions within disordered regions, translating a local chemical change into altered binding behavior [100, 101].

Beyond individual residue accessibility, multivalency represents a second structural principle that shapes PRMT substrate recognition. Many PRMT substrates, particularly RNA‐binding proteins such as heterogeneous nuclear ribonucleoprotein (hnRNP) family members, contain arginine‐rich low‐complexity regions that provide multiple potential methylation sites within flexible substrate contexts [102, 103]. Proteome‐wide studies show that arginine methylation is widespread across human proteins, suggesting that many PRMT substrates contain distributed methylation potential rather than a single isolated target site [8, 12]. In this view, structural flexibility determines how encoded arginine residues are presented to PRMTs, enabling repeated sampling of nearby candidate sites rather than recognition of a single rigid consensus motif. Therefore, multivalency bridges the sequence and structural layers by converting arginine‐rich motif density into the dynamic presentation of methylatable residues.

Finally, phase separation allows multivalent low‐complexity proteins, RNAs, and associated factors to segregate from the surrounding nucleoplasm or cytoplasm and form concentrated, dynamic biomolecular condensates, for as stress granules, processing bodies, and other ribonucleoprotein assemblies [104, 105, 106]. Although such condensates may spatially organize PRMT substrates and regulatory partners, whether they broadly enhance PRMT catalytic efficiency remains unclear. In the reverse direction, stronger evidence indicates that arginine methylation can regulate condensate behavior. In FUS and other RGG‐rich proteins, methylation weakens arginine‐dependent cation‐π interaction networks, reduces phase‐separation propensity, and alters condensate material properties [100, 101]. Proteome‐wide dimethylarginine profiling further extends this connection by linking arginine methylation to proteins involved in phase separation [12]. In stress granules, PRMT1‐mediated methylation of UBAP2L modulates granule assembly, providing a direct example of methylation‐dependent regulation of ribonucleoprotein (RNP) condensates [107].

To summarize, the structural layer acts as a bridge between sequence‐encoded methylation potential and higher order molecular organization, determining how accessible arginine residues are presented, modified, and functionally integrated into dynamic protein–RNA assemblies.

### 5.3 Localization layer—Spatial encounter probability

While the sequence and structural layers define where methylatable arginine residues are encoded and how accessible they are, arginine methylation also requires spatial proximity between PRMTs and their substrates. In this sense, the localization layer acts as a spatial filter that determines which potential substrates are physically reachable by each enzyme. Rather than uniformly sampling all proteins in the cell, PRMTs are more likely to methylate substrates that reside within the same cellular compartment or molecular neighborhood.

PRMT family members show distinct localization and interaction biases that align with their functional specialization. PRMT3 is linked to cytoplasmic ribosome‐associated substrate pools through its divergence from PRMT1 in subcellular localization and its interaction with ribosomal protein S2 [47, 48]. PRMT8 demonstrates a more spatially confined case, as N‐terminal myristoylation directs the enzyme to the plasma membrane, linking its localization to phospholipase activity and membrane‐associated lipid/signaling environment [59, 108, 109]. PRMT6 provides a nuclear/chromatin‐biased example, methylating histone H3R2 and H2AR29 within chromatin‐associated regulatory contexts [110, 111].

The contrast between PRMT1 and PRMT6 further illustrates how spatial determinants diversify substrate access even among closely related Type I enzymes. Although both enzymes share similar catalytic architectures, PRMT6 has been characterized as a nuclear enzyme with unique substrate specificity [56]. Beyond canonical histone substrates, PRMT6 has also been linked to chromosome‐associated regulatory outputs, including Aurora B positioning through H3R2me2a and RCC1‐dependent mitotic control [58, 112]. By contrast, PRMT1 engages a broader histone and nonhistone substrate landscape, including H4R3 and RNA‐associated regulatory proteins identified by methylome profiling [8, 103, 113]. This comparison supports the view that similar catalytic capacity can be routed toward distinct substrate pools when localization determinants and compartment‐specific substrate availability differ.

Importantly, localization should be viewed as a dynamic and bi‐directional layer of PRMT regulation, rather than a fixed upstream constraint. Notably, cellular stress and DNA damage can alter PRMT recruitment or compartmental distribution, thereby changing the substrate pools available for methylation. For instance, PRMT1 is recruited to chromatin by actions of DNA‐dependent protein kinase in response to cisplatin treatment, where it sustains senescence‐associated transcriptional programs through mediating H4R3me2a [114]. Reciprocally, arginine methylation can reshape the spatial dynamics of substrates themselves. Specifically, PRMT1‐dependent methylation regulates MRE11 nuclear compartmentalization, while arginine methylation controls the subcellular localization and splicing‐related functions of SF2/ASF, suggesting that methylation can redirect substrates toward distinct compartments or molecular assemblies [7, 115]. Together, the localization layer organizes PRMT–substrate encounter patterns, while methylation‐dependent changes in substrate localization can further remodel this spatial network according to cellular state.

### 5.4 Environmental layer—Physicochemical and cellular context

Even after sequence, structural, and localization requirements are met, arginine methylation remains sensitive to the surrounding cellular environment. PRMTs use SAM as the methyl donor and generate S‐adenosylhomocysteine (SAH) as a byproduct, associating catalytic output to methyl donor availability and methionine‐cycle metabolism. This relationship is particularly highlighted by methylthioadenosine phosphorylase (MTAP)‐loss contexts, where altered methionine metabolism creates dependence on PRMT5 activity [16]. Additionally, local physical conditions may influence substrate engagement by modulating weak electrostatic and multivalent interactions. The clearest evidence comes from phase‐separated systems, where arginine methylation alters cation‐π interaction networks and condensate behavior, while dimethylarginine profiling links methylation to phase‐separation‐associated proteins [12, 100, 101]. Cellular stress can also reshape PRMT output by altering enzyme abundance, stability, or recruitment to specific transcriptional programs. For example, nutrient stress activates an AMPK‐SKP2‐CARM1 signaling cascade that stabilizes CARM1, increases H3R17 methylation, and promotes transcriptional activation of autophagy and lysosomal genes, illustrating how environmental cues can redirect arginine methylation toward stress‐adaptive chromatin programs [116]. Finally, substrate abundance and macromolecular organization can tune methylation output, as PRMTs act within crowded substrate pools and protein complexes rather than on isolated targets [8, 15, 17]. Combinedly, the environmental layer modulates the efficiency and extent of arginine methylation by integrating metabolic state, local physicochemical conditions, and substrate organization.

## 6 Beyond catalysis: Nonenzymatic functions of PRMTs in cancer and the rationale for targeted degradation

PRMTs are traditionally viewed as catalytic enzymes; however, this perspective may not fully encompass the breadth of their functional roles. Most small‐molecule PRMT inhibitors are designed to bind the catalytic pocket—typically by competing with the SAM cofactor or substrate binding—rather than eliminating the PRMT protein itself [3, 41]. Meanwhile, emerging evidence suggests that certain PRMT functions may depend on their physical presence within macromolecular assemblies. For instance, CARM1 and PRMT5 function within multiprotein complexes that coordinate transcriptional regulation and RNA processing, raising the possibility that their regulatory functions may also depend on protein–protein interactions or structural roles within these assemblies [53, 117]. Moreover, PRMTs are embedded within regulatory networks involving protein complex stabilization, cofactor coordination, and interaction network organization [2, 11]. In sum, these findings suggest that the roles of PRMTs in cellular regulation may not be fully explained by enzymatic activity alone, but also reflect their integration within higher order molecular assemblies.

PRMT2 and CARM1 illustrate a related principle at a more general structural level. PRMT2 contains a regulatory SH3 domain and has relatively weak intrinsic methyltransferase activity, yet purified biochemical systems show that it can modulate PRMT1‐dependent histone methylation as a non‐catalytic component of the writer complex [118]. CARM1 also shows a comparable duality in cell‐cycle control: it can act as a scaffold linking CDK1 to the SKP2/Cullin‐1 ubiquitin ligase complex, thereby regulating nuclear CDK1 stability and contributing to the timing of mitotic entry independently of its methyltransferase output [119]. Together, these examples indicate that non‐catalytic domains and physical scaffolding interactions can shape PRMT‐dependent regulatory output alongside catalytic activity.

This distinction has particular relevance in cancer, where catalytic inhibition can suppress the methylation flux, but may leave intact oncogenic functions that depend on the continued physical presence of the PRMT proteins. Among PRMT family members, PRMT5 provides one of the clearest cancer‐relevant examples of catalysis‐independent regulation, and its scaffolding functions recur across different tumor contexts. In triple‐negative breast cancer, PRMT5 acts as a glucocorticoid receptor‐associated transcriptional scaffold, recruiting phospho‐HP1γ and RNA polymerase II to a subset of glucocorticoid‐responsive target genes and promoting cell migration in a manner reported to be independent of its methyltransferase activity [120]. A related coactivator function has also been described in prostate cancer, where early work suggested that PRMT5 can enhance androgen receptor transcriptional activity independently of full catalytic activity [121]. Together, these studies suggest that PRMT5 can support oncogenic transcription not only by depositing symmetric arginine methylation but also by helping organize transcriptional and cofactor complexes. PRMT1 illustrates a related principle at the level of substrate stabilization rather than transcriptional scaffolding. PRMT1 binds the orphan nuclear receptor TR3/Nur77 directly and delays its degradation without detectably methylating it [122]. This catalysis‐independent interaction is sufficiently specific that a hydrophobic‐tagging degrader, CM112, was later developed to deplete PRMT1 and thereby destabilize TR3, providing a chemical tool to probe this nonenzymatic function directly [123]. A related substrate‐level example has been reported in non‐small‐cell lung cancer, where PRMT1 and PRMT5 bind the anti‐apoptotic protein CFLARL/c‐FLIPL and exert opposing effects on its stability through differential engagement of the E3 ligase ITCH [124]. The finding that catalytically inactive mutants of both enzymes retain the ability to regulate CFLARL supports a model in which physical binding, rather than methylation alone, contributes to CFLARL turnover control [124].

Recognition of catalysis‐independent PRMT functions provides a rationale for therapeutic strategies that go beyond conventional active‐site inhibition. Catalytic inhibitors are well‐suited to suppress methyltransferase activity and reduce methylation flux, but they may leave intact scaffolding, adaptor, or complex‐stabilizing functions that depend on the physical presence of the PRMT protein. In settings where these noncatalytic functions contribute to malignant cell state maintenance, removal of the PRMT protein itself may provide a means to disrupt regulatory functions that are retained despite catalytic inhibition. Here, targeted protein degradation strategies, such as proteolysis‐targeting chimeras (PROTACs), provide a complementary approach to interrogate PRMT function by eliminating the entire protein rather than merely inhibiting its enzymatic activity [125]. Recent studies have successfully developed degraders across multiple members of the PRMT family, including PRMT1, PRMT3, CARM1, and PRMT5, collectively demonstrating that targeted degradation is a broadly applicable strategy within this enzyme class. Notably, these efforts have uncovered distinct functional insights depending on the PRMT subtype, ranging from noncatalytic stabilization (PRMT1) and phenotype divergence (PRMT3) to complex‐level regulation (PRMT5 and CARM1), suggesting that PRMT function extends beyond enzymatic activity in a context‐dependent manner. By removing the entire protein, PROTAC‐based degradation enables disruption of both catalytic and noncatalytic functions, including scaffold‐like roles that are otherwise retained under inhibition [126, 127]. Accordingly, this approach provides a unique experimental framework to distinguish whether a given phenotype is driven by methyltransferase activity or by the broader structural role of the PRMT protein.

Early efforts to develop PRMT1 degraders highlight the challenges in targeting PRMT1 for proteasomal degradation, as efficient degradation could not be achieved despite successful target engagement and recruitment of VHL or cereblon (CRBN) E3 ligases [128]. In light of these limitations, Ma et al. adopted an alternative strategy based on hydrophobic tagging, in which a PRMT1 inhibitor scaffold was conjugated to an adamantane moiety, termed CM112 [123]. This design bypasses the need for E3 ligase recruitment and enables proteasome‐dependent degradation of PRMT1 directly. More recent advances developed a bona fide PROTAC degrader by linking a PRMT1 inhibitor scaffold to a CRBN‐recruiting ligand, enabling ubiquitin/proteasome‐mediated degradation of PRMT1 [129]. In particular, the dimerization arm of PRMT1 plays a key role in facilitating ternary complex formation, underscoring the contribution of noncatalytic domains to protein–protein interactions. Similarly, a selective PRMT3 degrader was developed using an MDM2‐recruiting PROTAC, leading to depletion of PRMT3 and inhibition of its catalytic activity [130]. Notably, PRMT3 degradation resulted in stronger growth inhibition than SGC707‐mediated catalytic blockade and exhibited a synergistic antiproliferative effect when combined with the glycolysis inhibitor 2‐deoxy‐D‐glucose, suggesting a broader impact on cellular metabolism. Degraders targeting CARM1 are VHL‐recruiting PROTACs, yet vary in their warhead structure adopting either TP‐064 or EZM2302 [131, 132]. Strategies consistently show that CARM1 degradation suppresses tumor‐associated phenotypes, for as tumor growth *in vivo*, in a manner not fully recapitulated by catalytic inhibition, highlighting functional roles beyond its enzymatic activity. Within the PRMT degrader landscape, PRMT5 stands out as the most extensively developed target, with multiple PROTAC designs showing how changes in degrader architecture can reshape cellular efficacy. This progression began with the VHL‐recruiting degrader MS4322, which provided an early demonstration that PRMT5 could be eliminated through the ubiquitin–proteasome system, although its degradation potency and kinetics were not yet optimal [133]. Subsequent VHL‐based optimization yielded MS115, a more effective degrader capable of reducing the PRMT5–MEP50 complex in breast and prostate cancer models [134]. In parallel, CRBN‐directed designs further advanced the field, with YZ‐836P achieving low‐nanomolar degradation activity and antitumor efficacy in triple‐negative breast cancer organoid and xenograft models [135]. Taken together, these studies place PRMT5 at the forefront of PRMT degrader development and provide a strong precedent for expanding targeted degradation strategies across this enzyme family.

Despite this progress, targeted degradation does not overcome all limitations associated with the persistence of arginine methylation. PRMT degradation can halt further methylation while additionally removing noncatalytic functions that depend on the physical presence of the enzyme. However, removal of the PRMT protein is not expected to directly erase methylarginine marks already present on pre‐existing substrates, particularly in the absence of a broadly acting arginine demethylase. One potential future strategy may therefore be to combine PRMT degradation with approaches that promote turnover of persistent methylated substrate pools. Although this possibility has not yet been explored for PRMT‐regulated substrates, conceptually related approaches have been investigated in other disease settings. For example, activation of the valosin‐containing protein/p97 pathway by SMER28 has been reported to enhance autophagic and proteasomal clearance of pathogenic proteins in neurodegenerative models [136]. While such findings do not establish that methylarginine‐marked substrates can be selectively cleared through the same mechanism, they provide a conceptual precedent for combining targeted removal of a disease‐relevant regulator with broader enhancement of protein clearance. Whether analogous strategies could accelerate depletion of persistent methylated substrate pools following PRMT degradation remains an open question.

## 7 PRMTs in cancer: Competitive substrate allocation and stabilization of malignant states

Arginine methylation is not distributed uniformly across the proteome. Instead, proteomic and biochemical studies have consistently identified strong representation of RNA‐binding and processing proteins among arginine‐methylated substrates, along with numerous targets involved in transcription, chromatin regulation, signaling, and metabolism [1, 2, 8, 9, 10]. Notably, many of these substrates contain arginine‐rich or RG/RGG‐containing low‐complexity regions and intrinsically disordered segments that can provide permissive environments for PRMT engagement and multisite methylation [8, 84, 85]. However, sequence compatibility alone does not account for the broader diversity of cancer‐relevant PRMT substrates as discussed above [11, 22].

**Fig. 4:**
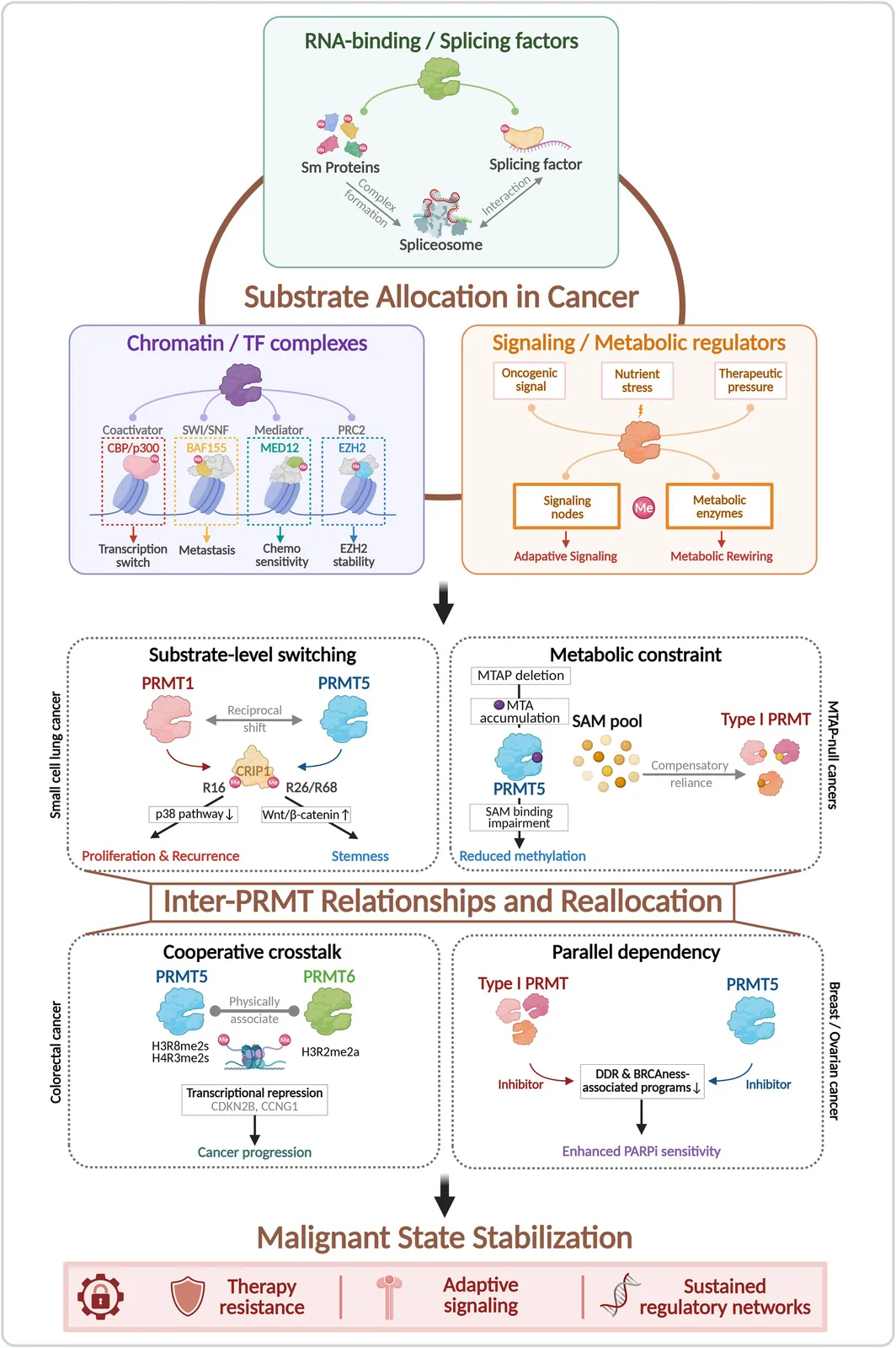
Inter‐PRMT relationships and substrate reallocation in malignant‐state stabilization. PRMT substrates implicated in cancer include proteins involved in RNA processing, chromatin/transcriptional regulation, and signaling/metabolic pathways (upper panel). Inter‐PRMT competition, compensation, cooperation, and parallel dependencies (middle panel) can influence substrate allocation and regulatory network activity, potentially contributing to malignant‐state maintenance (lower panel). BAF155, BRG1/BRM‐associated factor 155; CARM1, coactivator‐associated arginine methyltransferase 1; CBP, CREB‐binding protein; CCNG1, cyclin G1; CDKN2B, cyclin‐dependent kinase inhibitor 2B; CRIP1, cysteine‐rich intestinal protein 1; DDR, DNA damage response; EZH2, enhancer of zeste homolog 2; MED12, mediator complex subunit 12; MTA, methylthioadenosine; MTAP, methylthioadenosine phosphorylase; PARPi, poly(ADP‐ribose) polymerase inhibitor; PRC2, Polycomb repressive complex 2; PRMT, protein arginine methyltransferase; SAM, S‐adenosylmethionine; SWI/SNF, switch/sucrose non‐fermentable; TF, transcription factor. Created with BioRender.com. https://www.biorender.com/j2ay3vv.

In malignant cells, the functional substrate landscape may be further shaped by changes in molecular‐complex assembly, subcellular organization, metabolic state, and cellular stress [16, 17, 114, 116]. These features can alter which PRMT‐compatible proteins are available for productive enzyme‐substrate engagement, potentially shifting the relative contribution of different substrate pools without necessarily requiring changes in PRMT expression. We therefore examine PRMT function in cancer through the lens of substrate allocation: the context‐dependent engagement of RNA‐processing, chromatin and transcriptional, signaling and metabolic substrates within an interconnected methylation network. From this perspective, individual methylation events may collectively support broader regulatory programs that contribute to malignant cell‐state maintenance and adaptation (Fig. 4).

### 7.1 Cancer‐relevant PRMT substrate classes

To examine how this allocation logic operates across cancer‐relevant substrates, we broadly organize representative PRMT targets into three functional classes: RNA‐binding and splicing regulators, chromatin and transcriptional regulators, and signaling and metabolic proteins. Each category highlights different determinants of PRMT engagement, ranging from sequence and structural compatibility to recruitment within regulatory assemblies and cellular‐state‐dependent accessibility.

#### 7.1.1 RNA‐binding and splicing factors

RNA‐binding and splicing regulators constitute a prominent class of PRMT substrates and are recurrently represented across arginine‐methylation dataset [8, 12]. Intriguingly, many hnRNPs and other RNA‐binding proteins contain arginine‐rich or RG/RGG‐containing low‐complexity regions that provide permissive sequence‐level context for PRMT recognition and multisite methylation, whereas spliceosomal proteins are also frequently identified among PRMT substrates [84, 85]. Within the pICln‐containing complex, PRMT5 symmetrically dimethylates arginine residues in the C‐terminal RG‐rich tails of SmD1 and SmD3, enhancing their affinity for the SMN complex during snRNP assembly [90, 91]. Consistent with the central position of this pathway in spliceosome assembly, PRMT5 perturbation produces broad constitutive and alternative splicing defects across several cancer‐relevant settings [137, 138, 139].

PRMT‐dependent methylation also regulates other RNA‐processing factors beyond the Sm machinery. As an example, PRMT9 methylates the core spliceosomal factor SAP145/SF3B2, directly linking a Type II PRMT to U2 snRNP‐associated splicing [54, 61]. Arginine methylation of SRSF1/SF2‐ASF at R93, R97, and R109 regulates its nucleocytoplasmic distribution, with consequent effects on alternative splicing [115]. hnRNPA1 further illustrates how methylation of a single RNA‐binding protein can generate distinct functional outputs, as its methylation contributes to RNA binding, alternative splicing, and growth of various cancer cells [103]. In addition, PRMT5‐dependent methylation enhances hnRNPA1 binding to the IRESs of cancer‐relevant transcripts such as MYC and CCND1, thereby promoting their cap‐independent translation [140].

Many methylatable RNA‐binding proteins assemble into dynamic ribonucleoprotein compartments, placing PRMT substrates in environments where protein–protein and protein–RNA interactions are remodeled [104, 105, 106]. In the cytoplasm, PRMT1 asymmetrically dimethylates the RGG motif of UBAP2L, and increased methylation reduces its interaction with stress‐granule components, thereby suppressing granule assembly [107]. Within the nucleus, CARM1 promotes the export of IRAlu‐containing transcripts by methylating p54nrb to weaken its binding to these RNAs and by suppressing NEAT1 transcription to limit paraspeckle formation [141]. A complementary form of higher order organization occurs at the enzyme level. Disrupting PRMT1 oligomerization weakens its engagement with RGG‐rich RNA‐binding proteins, reduces their asymmetric methylation, perturbs RNA processing and splicing, and suppresses pancreatic tumor growth [86]. Overall, these findings suggest that RNA‐binding and splicing regulators form a high‐density and spatially organized substrate pool through which PRMTs can influence RNA processing, translational control, and adaptive RNP dynamics in malignant cells.

#### 7.1.2 Chromatin and transcriptional regulators

Chromatin and transcriptional regulators constitute another major class of PRMT substrates. In this class, substrate engagement appears to depend not only on sequence‐level preference but also on recruitment within specific transcriptional and chromatin‐associated assemblies.

Here, arginine methylation of histones provides the classical entry point into this regulatory layer. Representative examples are PRMT1‐mediated H4R3 methylation and CARM1‐mediated H3 arginine methylation, both linked to transcriptional activation [4, 5, 6], whereas PRMT5‐dependent symmetric arginine methylation has been associated with transcriptional repression [142]. Beyond histone targets, several cancer‐relevant nonhistone PRMT substrates are embedded within complexes that regulate transcriptional state, chromatin accessibility, proliferative identity, metastatic behavior, or therapeutic response. For instance, methylation within the CBP/p300 KIX region by CARM1 impairs its association with the CREB activation domain and consequently suppresses cAMP‐responsive transcription, illustrating the pathway‐dependent effects of CARM1 [117]. CARM1‐mediated BAF155 methylation provides another example, altering switch/sucrose nonfermentable‐associated chromatin remodeling in ways that support tumor growth and metastatic dissemination [143]. A follow‐up study further showed that methylation of BAF155 particularly reassigns activity of BRD4‐associated enhancer toward malignant programs while dampening interferon‐responsive gene expression [144]. MED12, a component of the Mediator complex, is also modified by CARM1 with consequences for transcriptional regulation as well as chemosensitivity [145].

A similar principle also applies to repressive and lineage‐associated chromatin modules. Methylation of EZH2 by PRMT1 increases its stability, proliferative features, and metastatic ability in breast cancer, while PRMT5 and EZH2 cooperate at the CDKN2B locus in colorectal cancer, coupling symmetric arginine methylation to Polycomb‐dependent silencing [15, 88, 146]. Such examples suggest that methylation of individual chromatin regulators may serve as anchoring points through which proliferative or metastatic programs are reinforced. Notably, PRMTs further intersect with transcription factors and tumor suppressor pathways. Indeed, arginine methylation of E2F1 regulates its opposing proliferative and apoptotic functions, whereas PRMT5 loss impairs cell‐cycle progression and attenuates p53‐dependent tumor‐suppressive responses [147, 148, 149].

Together, these examples indicate that PRMT‐dependent regulation extends beyond histone and chromatin enzymes to transcription factors and other components of regulatory complexes. Through these context‐dependent interactions, arginine methylation can influence coactivator activity, chromatin remodeling, transcriptional repression, and transcription‐factor output, thereby contributing to cancer‐associated transcriptional programs and malignant cell‐state maintenance.

#### 7.1.3 Signaling and metabolic regulators

Signaling and metabolic regulators constitute a third cancer‐relevant substrate class, in which site‐ and enzyme‐specific methylation can reshape pathway output and metabolic fitness [2, 3, 22]. EGFR illustrates this dependence within a single substrate: PRMT1‐mediated methylation of R198/R200 enhances ligand responsiveness and confers cetuximab resistance in colorectal cancer, whereas PRMT5‐mediated methylation of R1175 promotes Tyr1173 autophosphorylation while attenuating downstream ERK activation [150, 151]. Site‐specific methylation of other signaling regulators, including SMAD7 in TGF‐β signaling, AKT, and YBX1 upstream of NF‐κB, further illustrates how PRMTs act on components of distinct oncogenic pathways [152, 153, 154].

Metabolic substrates further illustrate that cancer‐associated arginine methylation is not uniformly increased. In liver cancer, elevated PHGDH R236 methylation can compensate for reduced PHGDH abundance by enhancing catalytic activity, serine production, and redox homeostasis, with higher methylation associated with increased enzyme activity and poorer patient outcome [155]. Comparable activating effects have been reported for PGK1, LDHA, and ENO1, whose site‐specific methylation enhances glycolytic activity in colorectal, hepatocellular, and lung cancers, respectively [156, 157, 158]. Conversely, CARM1‐dependent GAPDH R234 methylation suppresses glycolysis and tumor cell proliferation, yet this inhibitory mark is diminished in human hepatocellular carcinoma relative to normal tissue [159]. Together, these examples indicate that signaling and metabolic substrates form a context‐sensitive PRMT substrate pool, where enzyme identity and modification site shape distinct and sometimes opposing effects on tumor‐associated signaling and metabolism.

### 7.2 Inter‐PRMT relationships and substrate reallocation in malignant states

The substrate pools discussed above suggest that PRMT function in cancer is not simply the sum of independent enzyme‐substrate reactions. Rather, malignant cells may rely on a balanced arginine‐methylation network in which different PRMTs act on overlapping, adjacent, or functionally connected substrate modules. Since different types of PRMTs generate distinct methylarginine products while sharing the same methyl‐donor economy, PRMT‐dependent regulation may involve competition and compensation among enzyme pathways rather than isolated substrate selection alone.

When one PRMT pathway is inhibited, metabolically constrained, or displaced from a regulatory complex, alternative methylation routes or PRMT dependencies may become more prominent. This does not imply that PRMTs are fully redundant; rather, it suggests that partial overlap in substrate access or pathway function may generate context‐dependent dependencies between PRMT classes. A direct example of substrate‐level reallocation has recently been described in small‐cell lung cancer, where chemotherapy induces a sequential and reciprocal switch between PRMT1‐ and PRMT5‐mediated methylation of the same substrate, CRIP1. This switch is controlled by PELI1, an E3 ligase activated downstream of inflammatory signaling, and functionally promotes tumor recurrence after cisplatin and etoposide treatment, providing an example in which therapy‐induced inflammatory signaling redirects a single substrate between competing PRMT pathways [160]. The clinical relevance of this competitive logic is further underscored by MTAP‐deleted cancers, in which altered metabolite availability directly constrains PRMT5 activity rather than serving as passive metabolic background. Loss of MTAP elevates intracellular MTA, imposing a metabolic constraint on PRMT5 through competition with its methyl donor SAM [16, 23]. By reducing PRMT5‐dependent methylation capacity, this metabolic lesion partially disables PRMT5 function so that tumor cells may become more dependent on parallel or compensatory PRMT pathways, including type I PRMT activity. Consistent with this model, loss of PRMT1 increases cellular sensitivity to PRMT5 blockade, and pharmacological inhibition of type I PRMTs can synergize with PRMT5 inhibition in MTAP‐loss contexts, linking metabolic constraint on PRMT5 to compensatory dependence on the competing enzyme class [24, 161]. Importantly, this metabolically constrained PRMT5 state is not unique to MTAP loss. A genetically distinct route, LKB1 loss‐driven upregulation of nicotinamide N‐methyltransferase, can similarly lower the cellular SAM/SAH ratio and attenuate PRMT5 activity, thereby converging on a comparable metabolic vulnerability [162]. More recent therapeutic strategies, including MAT2A/PRMT5 co‐targeting and MTA‐cooperative PRMT5 inhibitors, further suggest that methylation capacity and PRMT pathway usage are shaped not only by enzyme expression but also by methyl‐donor availability and metabolite competition [94, 163, 164].

Additional cancer contexts further support the broader idea that PRMT dependencies can be shaped by pathway crosstalk rather than by isolated enzyme–substrate reactions alone. In colorectal cancer, PRMT5 physically and functionally interacts with PRMT6, and the two PRMTs cooperate at the promoters of CDKN2B and CCNG1 through PRMT6‐mediated H3R2me2a and PRMT5‐mediated H4R3me2s/H3R8me2s deposition. This results in transcriptional repression of these tumor suppressor genes and further drives colorectal tumor growth [165]. Meanwhile, in ovarian and breast cancer models with intact homologous recombination, blockade of either type I PRMTs or PRMT5 increases responsiveness to poly(ADP‐ribose) polymerase inhibitor, accompanied by reduced expression of DNA‐repair programs and greater treatment‐induced DNA damage [166]. Together with the CRIP1 and MTAP‐MTA‐PRMT5 examples above, these cases suggest that PRMT crosstalk may arise through multiple mechanisms, including substrate‐level switching, metabolic constraint, cooperative histone‐mark deposition, and parallel pathway dependencies.

These observations together suggest that cancer cells may depend on PRMTs not only because a single enzyme modifies a single oncogenic substrate but because multiple PRMT pathways collectively maintain RNA‐processing programs, chromatin‐regulatory complexes, signaling outputs, and metabolic adaptations. Under stress or therapeutic pressure, inhibition of one PRMT axis may shift methylation‐dependent regulatory load onto another axis, exposing hidden vulnerabilities. In this sense, PRMTs may function as a distributed methylation network in cancer, where malignant cell states are stabilized by the ability of PRMT pathways to compete, compensate and reallocate substrate engagement across changing cellular contexts.

Inter‐PRMT competition and substrate reallocation shift the therapeutic question from whether a given PRMT is simply overexpressed to how dependent a malignant cell state is on a constrained methylation network. Future PRMT‐targeting strategies may therefore need to consider not only catalytic inhibition of individual enzymes but also methylation‐capacity allocation across substrate pools, compensatory PRMT usage, methyl‐donor metabolism, and complex dependency. In some contexts, inhibition of a dominant PRMT axis may be sufficient; in others, residual methylation activity may persist through alternative PRMTs or preserved PRMT recruitment within regulatory assemblies. Rather than favoring a single therapeutic modality, this framework supports a context‐guided approach in which catalytic inhibitors, metabolic co‐targeting, combination strategies, or targeted degradation are evaluated according to how each perturbs the methylation network sustaining a given malignant state.

## 8 Conclusion

Arginine methylation has become increasingly recognized as a regulatory modification with diverse roles in cellular organization and disease biology. Studies of the PRMT family have further revealed that methylation output is shaped not only by catalytic specificity but also by substrate sequence, structural accessibility, subcellular localization, metabolic state, and association with macromolecular complexes [1, 3]. These regulatory layers provide a basis for understanding how arginine methylation influences RNA metabolism, chromatin organization, transcriptional control, signaling, and metabolic adaptation across cellular contexts [11, 22].

In cancer, PRMTs are linked to processes that support malignant progression, including altered RNA processing, transcriptional plasticity, chromatin remodeling, therapeutic adaptation, and metabolic rewiring [11, 20, 21, 155]. The evidence discussed in this review suggests that these effects are context‐dependent and may reflect the organization of PRMT‐compatible substrate pools rather than the activity of individual enzymes alone. Substrate availability, inter‐PRMT compensation and cooperation, methyl‐donor metabolism, and noncatalytic participation in regulatory complexes may therefore contribute to the emergence of PRMT dependencies in malignant cells [16, 23, 24, 161].

The regulatory ratchet framework proposed here offers one way to integrate these observations. Arginine methylation should not be interpreted as absolutely irreversible, nor should persistence of an individual methylarginine mark be equated with irreversible maintenance of a malignant phenotype. Instead, PRMT‐dependent regulation may promote state persistence through multiple kinetic and network‐level mechanisms, including stabilization of long‐lived methylated substrates, continuous methylation flux through renewed substrate pools, scaffold‐like PRMT functions, and inter‐PRMT compensation or cooperation. Together, these mechanisms may promote the stabilization of regulatory states in malignant cells while creating vulnerabilities when methylation capacity, substrate engagement, or PRMT‐containing complexes are disrupted.

Further studies integrating substrate‐resolved proteomics, structural and spatial analyses, chemical perturbation, and targeted degradation should help distinguish catalytic effects from broader functions of PRMT proteins within regulatory assemblies [3, 8, 15, 129, 130, 131, 132, 133, 134, 135]. Therapeutic strategies may likewise need to consider substrate‐pool allocation, compensatory PRMT relationships, methyl‐donor metabolism, and catalysis‐independent functions rather than relying solely on enzyme expression or inhibition of individual PRMTs. Catalytic inhibitors reduce ongoing methylation, whereas targeted degradation may additionally remove PRMT protein‐dependent functions that do not require catalysis. As neither approach directly removes methylarginine already present on substrate proteins, the turnover kinetics of pre‐existing methylated substrates may remain an important determinant of response. Ultimately, this framework shifts attention from individual methyltransferase activities toward the methylation‐supported regulatory networks that sustain malignant cell states and the context‐specific vulnerabilities created by their disruption.

## Conflict of interest

The authors declare no conflict of interest.

## Author contributions

SHK and JML conceived the scope and conceptual framework of the review. SHK conducted the literature synthesis and drafted the original manuscript. JML supervised the work, acquired funding, and critically reviewed and edited the manuscript. Both authors read and approved the final manuscript.
